# AKT in Stromal Fibroblasts Controls Invasion of Epithelial Cells

**DOI:** 10.18632/oncotarget.1078

**Published:** 2013-07-14

**Authors:** Ann-Christin Cichon, Adam Pickard, Simon S. McDade, Daniel J. Sharpe, Michael Moran, Jacqueline A. James, Dennis J. McCance

**Affiliations:** ^1^ Centre for Cancer Research and Cell Biology, Queen's University, Belfast, UK

**Keywords:** stroma, invasion, AKT, Caspase 1, HPV, mdm2, interleukin 1, KGF, cancer

## Abstract

The tumour microenvironment has an important role in cancer progression and recent reports have proposed that stromal AKT is activated and regulates tumourigenesis and invasion. We have shown, by immuno-fluorescent analysis of oro-pharyngeal cancer biopsies, an increase in AKT activity in tumour associated stromal fibroblasts compared to normal stromal fibroblasts. Using organotypic raft co-cultures, we show that activation of stromal AKT can induce the invasion of keratinocytes expressing the HPV type 16 E6 and E7 proteins, in a Keratinocyte Growth Factor (KGF) dependent manner. By depleting stromal fibroblasts of each of the three AKT isoforms independently, or through using isoform specific inhibitors, we determined that stromal AKT2 is an essential regulator of invasion and show in oro-pharyngeal cancers that AKT2 specific phosphorylation events are also identified in stromal fibroblasts. Depletion of stromal AKT2 inhibits epithelial invasion through activating a protective pathway counteracting KGF mediated invasions. AKT2 depletion in fibroblasts stimulates the cleavage and release of IL1B from stromal fibroblasts resulting in down-regulation of the KGF receptor (fibroblast growth factor receptor 2B (FGFR2B)) expression in the epithelium. We also show that high IL1B is associated with increased overall survival in a cohort of patients with oro-pharyngeal cancers. Our findings demonstrate the importance of stromal derived growth factors and cytokines in regulating the process of tumour cell invasion.

## INTRODUCTION

Cancer is a disease of transformed epithelial cells but in recent years the importance of the stromal compartment in influencing the proliferation and invasive capacity of cancer epithelial cells, has become apparent. In normal stratified epithelium, Maas-Szabowski *et al*. suggested that the communication between the epithelium and the stromal fibroblasts is mainly based on the release of cytokines and growth factors, which are essential for epithelial maturation [1, 2]. They and others proposed that by releasing Interleukin-1alpha (IL1A) and Interleukin-1beta (IL1B), epithelial keratinocytes stimulate the release of Keratinocyte Growth Factor (KGF, also known as fibroblast growth factor 7, FGF7) by the stromal fibroblasts and KGF in turn, is required to influence keratinocyte differentiation and proliferation [2-4]. Further studies revealed, that as well as controlling keratinocyte differentiation and proliferation, KGF also regulates invasion of pre-malignant keratinocytes [5].

AKT can be activated in cells by reduced levels or mutations in Phosphatase and Tensin homology protein (PTEN) and increased activity through mutations of the subunits of Phosphatidylinositide 3-kinases (PI3 kinases) and it has recently been reported that loss of stromal PTEN dramatically increases mammary tumour formation in a MMTV-ErbB2 breast cancer model in mice [6, 7]. Also, the AKT pathway is modulated in the stroma of human breast cancers through mutation of PTEN and PI3K [8]. In addition, Pickard *et al*. showed that active AKT in the stromal fibroblasts correlated with increased invasive potential of HPV 16 E6 and E7 expressing, pre-malignant epithelium [5] AKT has three distinct isoforms, which even though they share a high percentage of homology are known to have individual functions [9]. Knockout mouse models demonstrate the diversity of each isoform, as AKT1 knockout mice exhibit severe growth defects and 40% die during the first weeks of life [10]. On the other hand, AKT2 knockout mice have deficiencies in glucose metabolism and adipogenesis [11, 12] and AKT3 knockout mice suffer from a deficiency in brain growth and development [13]. Although these mouse models might implicate a difference in tissue expression of the AKT isoforms, further studies have revealed roles for all three isoforms in regulating tumourigenesis in various tissues [14]. In spite of the growing importance of stromal PTEN and PI3 kinases, which isoform of AKT mediates these stromal effects is yet to be investigated.

Here we propose a role for stromal AKT2 in regulating the cross-talk between stroma and epithelium and thereby controlling invasiveness of potentially malignant epithelium. Our findings suggest that loss of PTEN function in stromal fibroblasts induces epithelial invasions in an AKT2 and KGF dependent matter, the latter growth factor promoting invasion. Depletion of AKT2 inhibits invasion but surprisingly does not alter KGF levels. However, AKT depletion does cause an increase in IL1B, which inhibits the function of KGF by reducing the expression of its receptor, FGFR2b, in tumour cells. The overall result is the inhibition of AKT2-mediated epithelial cell invasion.

## RESULTS

### AKT is activated in the stroma associated with Head and Neck cancers

**Figure 1:**
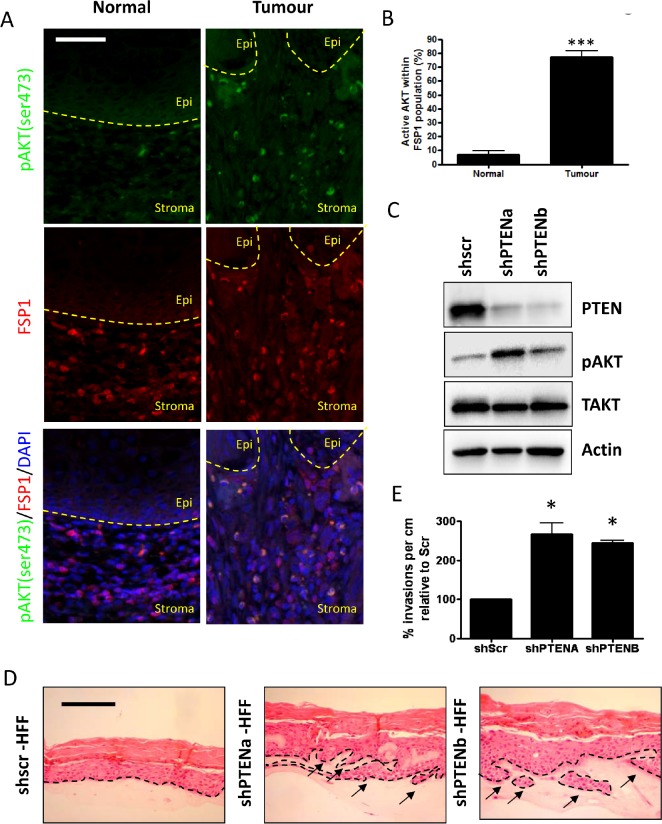
Stromal fibroblasts in oro-pharyngeal cancers contain activated AKT A) Oro-pharyngeal tumour samples were stained with anti-pAKT (Ser 473) and anti-Fibroblast Specific Protein 1 (FSP1) White arrows show cells staining positive for both pAKT (Ser 473) and FSP1. Scale bar = 25 µm. B) Quantification of staining, where cells were counted as follows: all FSP1 positive cells were counted (to a total of 600 cells per normal and tumour section each per slide). Cells stained for both pAKT (Ser 473) and FSP1 were also counted. The histogram shows the percentage of cells that were pAKT(Ser 473) and FSP1 positive out of all FSP1 positive cells. C) Knockdown of PTEN in primary human foreskin fibroblasts. Western blot analysis shows knockdown and subsequent increase in active, phosphorylated AKT at Ser 473. D) Organotypic raft cultures of scramble control and PTENa and PTENb knockdown fibroblasts cultured with E6/E7-expressing keratinocytes showing invasion of keratinocytes. Arrows distinguish the basal membrane. Scale bar = 100µm. E) Quantification of invasions per cm of raft in relation to the scramble control raft culture shows an induction of invasions after loss of PTEN in stromal fibroblasts. P-values: *=p<0.05; ***=p<0.001. All experiments were repeated 3 times.

To investigate whether stromal AKT activation is important in oro-pharyngeal tumours, matched normal and tumour samples from 34 oro-pharyngeal tumour samples were stained with pAKT (Ser 473) as a readout of AKT activity [15] and Fibroblast Specific Protein 1 (FSP1) as a marker for stromal fibroblasts (Figure 1A). From this the percentage of stromal fibroblasts containing active AKT was determined. Approximately 75% of FSP1 positive fibroblasts contained active AKT when associated with the tumour, a seven-fold increase compared to fibroblasts associated with the matched histologically normal epithelium (Figure 1B). A small proportion of pAKT staining in the stroma did not correlate with FSP1 staining, or with either vimentin (Supplemental Figure S1A and B), or smooth muscle actin-positive cells (data not shown) and may represent infiltrating immune cells.

### Expression of AKT in stromal fibroblasts is crucial for invasion of pre-malignant keratinocytes

To investigate the impact of stromal AKT activation on epithelial invasions, we used three-dimensional organotypic raft cultures, where human fibroblasts are placed in a collagen plug that acts as the stroma and keratinocytes seeded on top of the collagen as the epithelial layer. To activate AKT, we depleted PTEN levels in primary human foreskin fibroblasts (HFFs) using retrovirally expressed short-hairpin RNA (shRNA) and reduced PTEN protein levels were confirmed by western blot along with the corresponding activation of AKT, as shown by pAKT (Ser 473) levels (Figure 1C). To assess the effect of PTEN depleted fibroblasts on epithelial growth, primary human foreskin keratinocytes (HFK) were transformed with a retroviral vector expressing the Human Papilloma Virus (HPV) Type 16 E6 and E7 proteins, which results in immortalized but non-tumourigenic cells [5]. Organotypic raft cultures were grown for 14 days, after which they were fixed and Heamatocylin and Eosin (H + E) stained to determine the level of invasion. In raft cultures containing fibroblasts expressing a scramble control sequence(shSCR), keratinocytes infrequently invaded into the collagen plug (Figure 1D). However, using two different short hairpin RNAs (shRNA) to PTEN, we observed invasion of the immortalized keratinocytes in PTEN depleted fibroblasts (Figure 1D) that were subsequently counted and quantification (Figure 1E). These experiments have been carried out using three independently generated HFFs and E6/E7 expressing HFKs.

### AKT2 mediates invasions

To investigate if AKT was involved in stimulating invasion, we used a siRNA targeted to all three AKT isoforms. Depletion was confirmed by real-time PCR and Western blot (Supplemental Figure S2A and S2B) and the fibroblasts transferred into collagen plugs to examine their effect on pre-malignant keratinocyte invasions. By depleting PTEN knockdown fibroblasts of all three AKT isoforms, invasion of E6/E7-expressing keratinocytes was inhibited (Supplemental Figure S2C and S2D), confirming that the invasive phenotype mediated by PTEN knockdown fibroblasts is indeed an AKT dependent function.

**Figure 2:**
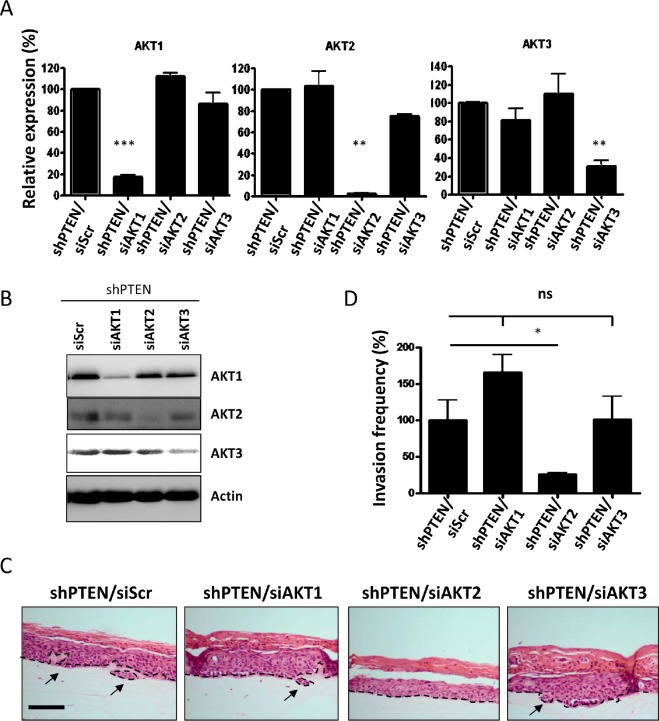
Depletion of individual AKT in PTEN knockdown HFFs PTEN knockdown fibroblasts were depleted of AKT1, AKT2 and AKT3 and knockdown was confirmed by A) real-time PCR analysis and B) Western blots. C) Organotypic raft co-cultures using E6/E7 expressing keratinocytes and PTEN depleted fibroblasts that were treated with either siRNA molecules directed against scramble control (siScr), AKT1 (siAKT1), AKT2 (siAKT) and AKT3 (siAKT). Scale bar = 100µm. Panels are representative of three separate experiments. D) Quantification of invasions per cm of raft in relation to the scramble control raft culture.

**Figure 3:**
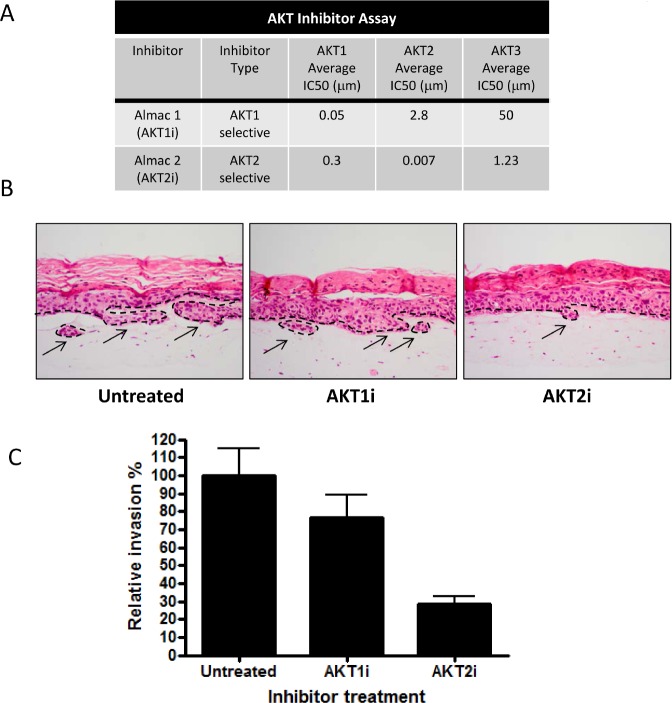
An AKT2 inhibitor reduces invasion A) The activity and specificity of inhibitors to AKT 1 and 2. B) Inhibitors specific for AKT1 (AKTi) and AKT2 (AKT2i) were used to treat organotypic cultures containing depleted PTEN HFFs. Scale bar = 100µm. C) Quantization shows that only cultures treated with the AKT2 inhibitor (AKT2i) had reduced invasion of E6/E7 expressing HFKs. P values: **=p<0.01. ns = not significant. All experiments were repeated 3 times.

AKT has three isoforms; AKT1, AKT2 and AKT3 and although their functions often overlap, they have been shown to have individual roles regarding invasion and metastasis [16, 17]. To establish whether this holds true in a cell non-autonomous fashion and to determine which isoform mediates invasion, AKT isoforms were knocked down in HFFs depleted of PTEN using individual short interfering RNA (siRNA) molecules. Knockdown of individual isoforms was confirmed using real-time PCR and Western blots (Figure 2A and B). Fibroblasts were then transferred into collagen plugs and used in organotypic raft cultures. Knockdown was retained after 14 days of culture as determined by real-time PCR (Supplemental Figure S3A-C), due to the fact that the fibroblasts in the collagen plug do not proliferate. Whilst knockdown of AKT1 and AKT3 did not alter keratinocyte invasions, knockdown of AKT2 significantly reduced keratinocyte invasions to levels of control rafts (Figure 2C and D). Hence, knockdown of AKT2 was able to inhibit stromal fibroblast mediated invasions, indicating that these invasions are AKT2 dependent. To further confirm the role of stromal AKT2 in mediating epithelial invasions, we also used specific inhibitors of AKT1 and AKT2 (Figure 3A) to show that only inhibition of AKT2 reduced the level of invasion of PTEN depleted rafts (Figure 3B and C). We also exogenously expressed HA-AKT isotypes and a catalytically inactive HA-AKT2 molecule in HFFs (Supplemental Figure S4A) and assessed epithelial invasion (Supplemental Figure S4B). Only expression of the active AKT2 was able to significantly increase invasion of the E6/E7-expressing keratinocytes (Supplemental Figure S4C).

**Figure 4:**
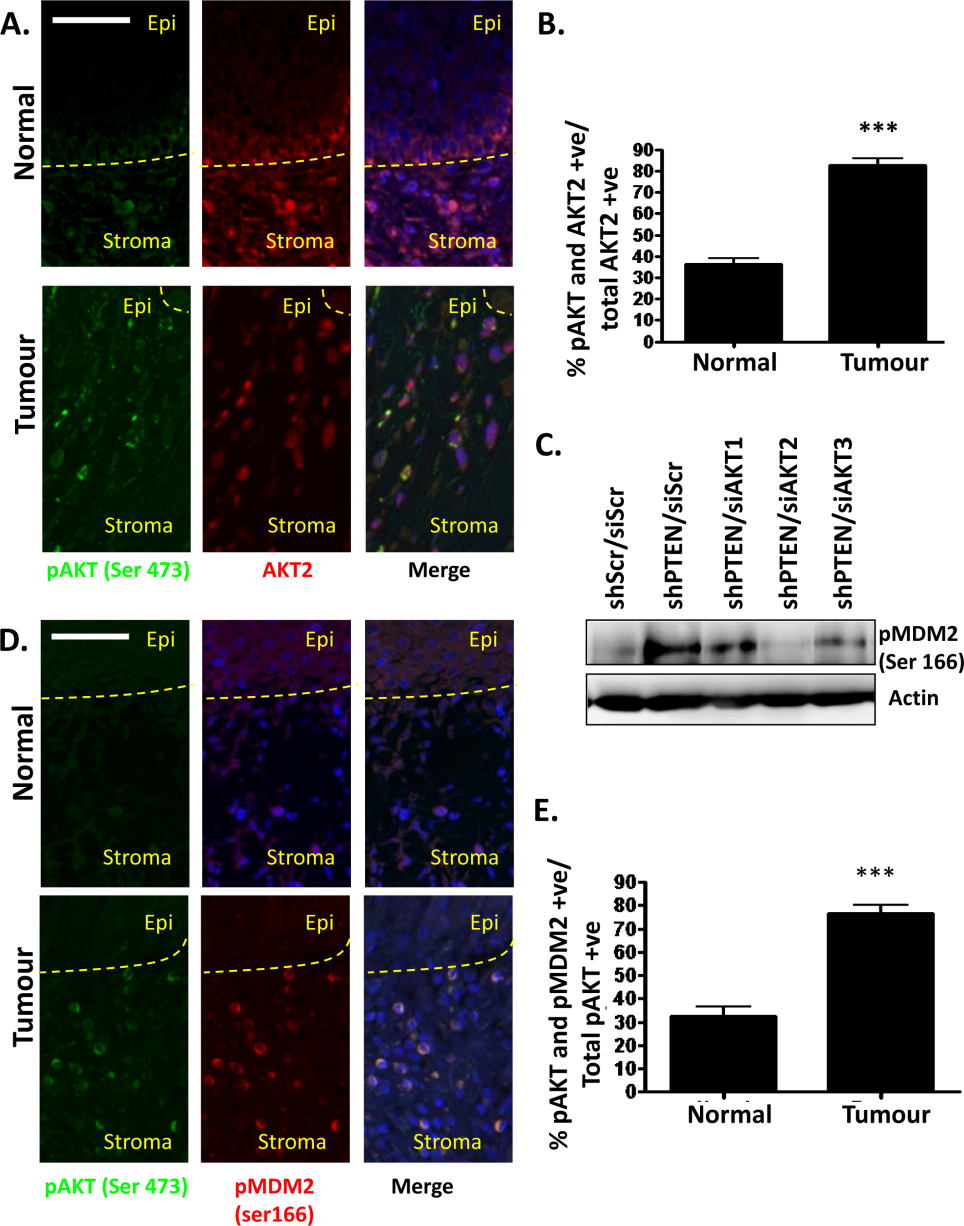
Stromal fibroblasts in oro-pharyngeal cancers exhibit activated AKT2 A) Oro-pharyngeal tumour samples were stained with anti-pAKT (Ser 473) and AKT2. Scale bar = 25 µm. B) The histogram shows quantification of staining, where cells were counted as follows: all AKT2 positive cells were counted (to a total of 600 cells per normal and tumour section each per slide). Cells that stained for both pAKT (Ser 473) and AKT2 were also counted. The histogram shows the percentage of cells that were pAKT (Ser 473) and AKT2 positive out of all AKT2 positive cells. C) PTEN knockdown fibroblasts were depleted of AKT1, AKT2 or AKT3. Western blot analysis shows pMDM2 (Ser 166) levels as an indicator of AKT2 activity. D) Oro-pharyngeal cancer biopsy sections were co-stained with pAKT (Ser 473) and pMDM2 (Ser 166) conjugated to a fluorophore. White arrows indicate representative cells stained with pAKT and pMDM2. Scale bar = 25 µm. E) Quantification of staining, where cells were counted as follows: all pAKT (Ser 473) positive cells were counted (to a total of 600 cells per normal and tumour section each per slide). Cells that stained for both pMDM2 (Ser 166) and pAKT (Ser 473) were also counted. The histogram shows the percentage of cells that were pMDM2 (Ser 166) and pAKT (Ser 473) positive out of all pAKT (Ser 473) positive cells. P-values: ***=p<0.001.

To investigate whether activation of stromal AKT2 may be important in tumourigenesis of oro-pharyngeal cancers, tumour sections were co-stained with antibodies raised against pAKT (Ser 473) and AKT2 (Figure 4A). Dual stained cells were counted in stroma associated with normal and tumour tissue and demonstrated a significant increase in the proportion of AKT2 positive cells, that were also positive for pAKT (Ser 473) in tumour associated stroma (Figure 4B). To further confirm this observation, we used an antibody, which detects the phosphorylation of MDM2 at the Serine 166 residue, as this had previously been reported to be an AKT2 specific phosphorylation event [18]. To confirm that MDM2 (Ser 166) was indeed an AKT2 specific phosphorylation site, cell lysates from PTEN and AKT isoform depleted fibroblasts were analysed by western blot analysis (Figure 4C). Phosphorylated MDM2- ser 166 is increased in PTEN depleted fibroblasts, which is then reduced upon additional knockdown of AKT2. This was not observed in cells where AKT1 or AKT3 were depleted, confirming that this site is AKT2 specific. Using pMDM2 (Ser 166) as an indicator of AKT2 activity matched normal and tumour oro-pharyngeal tumour sections were co-stained with pAKT (Ser 473) (Figure 4D) and this revealed a strong correlation between active AKT and pMDM2 (Ser 166) in tumour stroma compared to normal stroma (Figure 4E). These results indicate that AKT2 is activated in the stroma of oro-pharyngeal cancers, correlating with our findings in organotypic raft cultures.

We had previously shown that in fibroblasts depleted of the retinoblastoma protein (Rb), AKT was activated and induced invasion of E6/E7-expressing keratinocytes through a KGF-dependent mechanism [5]. To determine if invasion was mediated by KGF in PTEN depleted fibroblasts, we depleted KGF in PTEN knockdown cells and showed that, as with Rb depleted cells, invasion of epithelial cells was mediated by KGF (Supplemental Figure S5A-C).

### IL1B prevents KGF mediated invasions

**Figure 5:**
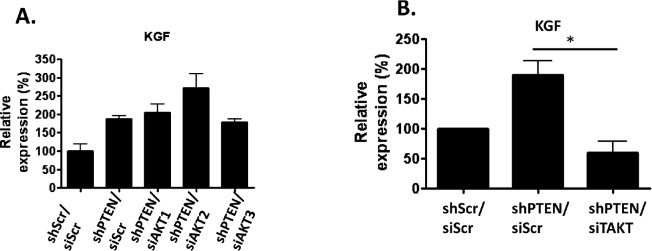
KGF mRNA levels in AKT depleted cells: A) Scramble control, PTEN, and PTEN and either AKT1, AKT2 and AKT3 knockdown fibroblasts were analyzed using real-time PCR analysis for KGF mRNA levels B) PTEN knockdown fibroblasts were depleted of all three AKT isoforms (shPTEN/siTAKT) and KGF mRNA levels were examined using real-time PCR.

Since AKT mediated invasions were KGF dependent, we next examined KGF levels in PTEN and AKT isoform depleted fibroblasts to determine if AKT2 activity was responsible for the increase in KGF that resulted in invasion. While there was an increase in KGF mRNA levels upon PTEN knockdown, KGF mRNA levels (Figure 5A) and protein (data not shown) were not significantly altered when AKT1, AKT2 or AKT3 where knocked down individually in addition to PTEN. However, KGF levels were reduced to control levels upon knockdown of all three isoforms simultaneously (Figure 5B), showing that KGF expression is AKT dependent, but that the AKT isoforms can possibly compensate for each other in the regulation of KGF expression. Compensation between isoforms has also been shown in AKT1/AKT2 double knockout mice, which suffer from severely impaired skin development, not seen in single knockout mice [19].

**Figure 6:**
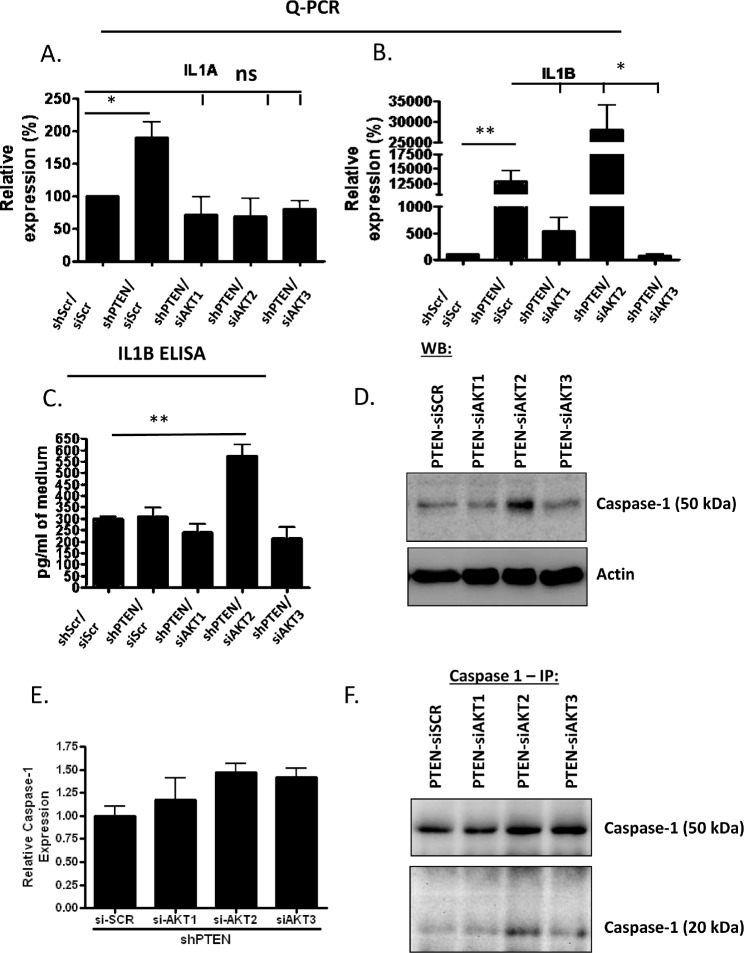
IL1B expression, cleavage and release in PTEN/AKT2 depleted fibroblasts A) IL1A levels were determined in fibroblasts depleted for PTEN and each single AKT isoform using real-time PCR. B) As in (A) but IL1B levels are shown. C) ELISA analysis for IL1B in media from organotypic cultures where the fibroblast cells were depleted of PTEN and individual AKT isoforms. Media was concentrated 14 times before ELISA was performed. Figure is average of two separate experiments. D) Western blot showing increase in caspase 1 in PTEN/AKT2 depleted fibroblasts. E) mRNA levels of IL1B show that expression of Caspase 1 is not significantly different in fibroblasts depleted of PTEN and each of the individual AKT isoforms. F) Shows the increase in the cleaved form of caspase 1 in PTEN/AKT2 depleted fibroblasts.

KGF is not the only growth factor/cytokine involved in the paracrine cross-talk between stromal fibroblasts and epithelium [2]. IL1A and IL1B have previously been shown to stimulate fibroblasts to produce KGF [1]. To establish IL1 levels in AKT2 depleted fibroblasts, the mRNA levels of both IL1A and IL1B were examined using quantitative real-time PCR. Similarly to KGF levels, an increase in IL1A levels was observed when PTEN was depleted in HFFs (Figure 6A). Knockdown of any of the AKT isoforms decreased IL1A levels to that of the scrambled control (Figure 5A). Examination of IL1B levels revealed that the increased IL1B levels observed in PTEN depleted fibroblasts were mediated by either AKT1 or AKT3, while depletion of AKT2 resulted in a further increase in IL1B (Figure 6B). ELISA confirmed this result, which also revealed, that the increase in IL1B levels detected in PTEN only knockdown fibroblasts (Figure 6B) did not result in increased secretion of the protein (Figure 6C). However, the increased mRNA expression levels upon further depletion of AKT2 in fibroblasts were translated into secreted IL1B protein (Figure 6C) and so implicate AKT2 in the regulation of IL1B translation/secretion. To investigate this further, we determined the level of caspase 1, Interleukin-1 cleaving enzyme (ICE), which cleaves IL1B and allows secretion of the active protein. Figure 6D shows that caspase 1 protein levels are elevated specifically in cells depleted of AKT2 and not in cells depleted of AKT1 or 3. This was not observed at the transcriptional level (Figure 6E), suggesting AKT2 regulates Caspase 1 levels via a post-transcriptional mechanism. Following immunoprecipitation of Caspase 1 from conditioned medium from these cells, the active fragment of Caspase-1 could be observed (Figure 6F). Therefore, the depletion of both PTEN and AKT2 resulted in increased expression and release of active IL1B, while the depletion of PTEN and AKT1 or 3 increased expression of IL1B without releasing the active form.

**Figure 7:**
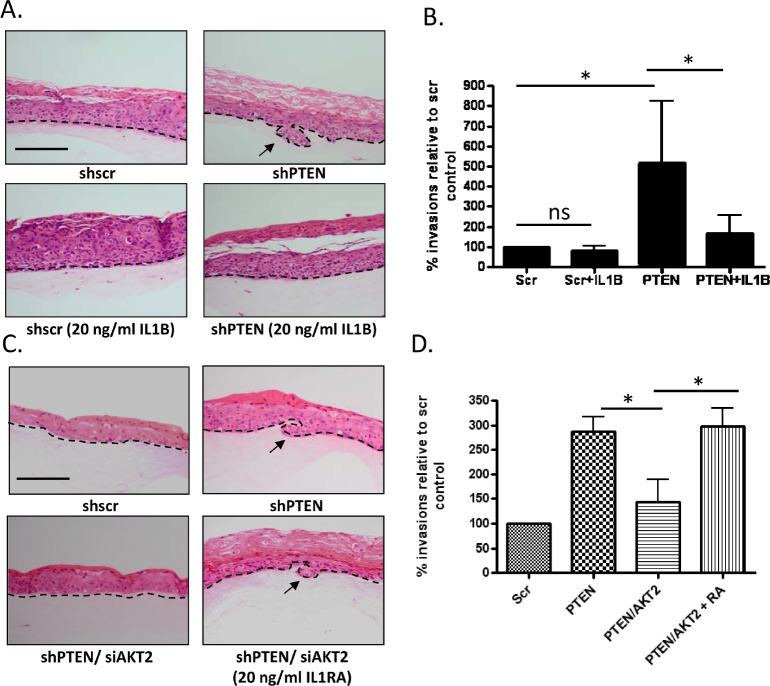
IL1B inhibits invasion A) Organotypic raft co-cultures using scramble control and PTEN knockdown fibroblasts and E6/E7-expressing keratinocytes. Raft cultures were treated with 20 ng/ml of IL1B. Scale bar = 100µm. Organotypic raft cultures are a representative of 3 biological replicates. B) Quantification of invasions into the collagen matrix per cm of raft culture from (A) relative to scramble control raft cultures from three independent experiments. C) Organotypic raft co-cultures using scramble control, PTEN knockdown fibroblasts and PTEN/AKT2 knockdown fibroblasts and E6/E7 expressing keratinocytes. PTEN/AKT2 fibroblasts raft cultures were treated with 20 ng/ml of IL1RA. Scale bar = 100µm. Organotypic raft cultures are a representative of 3 biological replicates. D) Quantification of invasions into the collagen matrix per cm of raft culture relative to scramble control raft cultures from three independent experiments. P value: *=p<0.05. ns=not significant.

We hypothesised that the increased levels of IL1B in the PTEN/AKT2 depleted cells might have a protective effect on the invasion process. To determine the effect of IL1B on invasion we treated organotypic raft cultures containing either scramble control or PTEN knockdown fibroblasts with 20 ng/ml of recombinant IL1B. The amount of IL1B used was determined based on previous reports investigating IL1B levels in healthy dental pulps [20]. Whilst IL1B had no effect on the epithelium grown with control fibroblasts, IL1B treatment of cultures grown with PTEN depleted HFFs resulted in a significant reduction in the number of invasive events (Figure 7A and B). This was further confirmed by treatment of PTEN/AKT2 depleted fibroblast cultures with the IL1 receptor antagonist (IL1RA), to sequester the secreted IL-1B and preventing binding to the IL1 receptor. Through blocking IL1B signalling keratinocyte invasion was induced (Figure 7C and D), indicating that IL-1B had an inhibitory effect on invasion. This could be specifically attributed to IL1B through knockdown of IL1B in PTEN/AKT2 double knockdown fibroblasts, which again induced keratinocyte invasion (Supplemental Figure S6A and B). These results demonstrated that AKT2 depletion activates a protective pathway, mediated by IL1B, which inhibits the pro-invasive KGF pathway and implies that AKT2 depletion leads to the inactivation of this normally protective pathway.

### IL1B inhibits epithelial FGFR2B expression and increases overall survival

**Figure 8:**
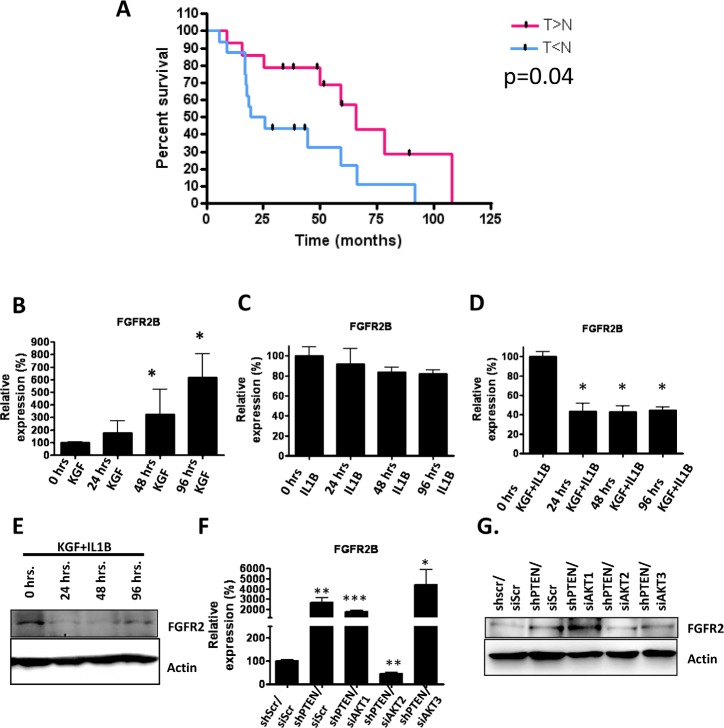
A) mRNA was obtained from 22 oro-pharyngeal tumour biopsies and matched non-tumour tissue and IL1B and FSP1 levels were determined using real-time PCR analysis Patients with high levels of IL1B in the tumour-associated stroma compared to the stroma associated with non-tumour tissue had a better overall survival. B) Real-time PCR analysis of FGFR2B expression levels after KGF treatment of E6/E7-expressing cells. C) Real-time PCR analysis of FGFR2B expression levels after IL1B treatment of E6/E7 expressing cells. D) Real-Time PCR analysis of FGFR2B expression levels after combined KGF and IL1B treatment of E6/E7-expressing cells. E) Western blot analysis confirms the decreased expression of FGFR2B from (C). F) PTEN knockdown fibroblasts were depleted of each AKT isoform using siRNA molecules and these fibroblasts were used in organotypic raft co-cultures containing E6/E7-expressing epithelia. The epithelium was peeled off each raft culture and FGFR2B mRNA measured by real-time PCR. G) Western blot analysis of epithelia examining FGFR2 protein expression. Western blot analysis is representative and real-time PCR analysis an average of 3 biological replicates. P values: *=p<0.05, **=p<0.01, ***=p<0.001.

IL1B inhibited invasion in our model system, so to determine if the levels of IL1B in oro-pharyngeal cancers had a protective effect, we examined the mRNA levels of IL1B by real-time PCR in matched non-tumour and tumour tissue from 22 oro-pharyngeal cancers. To take into account that these samples include a mixture of epithelial and stromal content, FSP1 expression was determined as a measure of the content of stroma. IL1B levels were normalised to ribosomal protein large P0 (RPLPO) and then to FSP1. The difference in the expression of IL1B in the tumour and matched non-tumour samples was calculated and correlated to patient overall survival. Enhanced expression of IL1B in the tumour-associated stoma compared to the stroma from matched non-tumour tissue showed a more favourable outcome than those with reduced expression of IL1B in the tumour associated stroma (Figure 8A).

Since IL1B inhibited invasion even in the presence of elevated KGF levels we investigated whether IL1B influenced KGF mediated signalling. We therefore investigated whether IL1B treatment altered the expression of FGFR2B. E6/E7 expressing keratinocytes were treated with KGF alone, IL1B alone, or a combination of KGF and IL1B. KGF treatment resulted in increased expression of FGFR2B mRNA levels over a 96-hour time course (Figure 8B), in comparison IL1B treatment did not alter the expression of FGFR2B (Figure 8C). However, when cells were treated with KGF in the presence of IL1B, there was no induction of FGFR2b expression in fact there was a significant reduction in FGFR2B levels in keratinocytes both at the level of mRNA and protein (Figure 8D and E). These data suggest that the IL1B signalling pathway can inhibit KGF signalling by reducing KGF dependent FGFR2B expression. To further evaluate this hypothesis in the organotypic culture model, the epithelia was removed from organotypic cultures grown in the presence of PTEN depleted fibroblasts plus each of the individual AKT isoforms and the expression of FGFR2B was examined by real-time PCR and western blot analysis. Epithelia cultured with PTEN/scr, PTEN/AKT1 and PTEN/AKT3 knockdown fibroblasts, showed an increase in FGFR2B expression compared to controls (scr/scr), whereas epithelia from PTEN/AKT2 knockdown fibroblast-containing stroma showed a decrease in FGFR2B expression to that of the controls (Figure 8F and 8G). Since we had previously shown that FGFR2B was required for invasion [5], these current results show that IL1B can inhibit invasion through down-regulation of the FGFR2B receptor.

## DISCUSSION

The work presented here, using 3D organotypic cultures and tumour tissue, support evidence indicating cell non-autonomous roles for AKT in tumourigenesis. We have shown AKT activation in primary human fibroblasts results in invasion of the epithelium and supports the recent findings in mouse breast tissue [7], where conditional PTEN knockdown in FSP1 expressing stromal fibroblast cells, resulted in enhanced tumour formation. We have gone on to show for the first time that stromal AKT function is required to regulate expression of KGF, a driver of epithelial invasion. Our findings are confirmed by analysis of microarray data from the stromal compartment associated with mammary tumours in mice with PTEN-depleted fibroblasts which also express elevated levels of KGF (GEO Dataset GSE16073, [7]). We have also observed that in stromal fibroblasts, AKT is activated in areas surrounding tumour epithelium in oro-pharyngeal cancers. Using organotypic raft cultures we have shown that the invasion induced by AKT activation is due to AKT2. This has been shown in three ways, firstly, depletion of AKT2, secondly, inhibition of invasion by an AKT2 specific inhibitor and thirdly, over-expression of AKT2. This work is also in agreement with experiments conducted in the cancer epithelium, where AKT2 has been shown to be a key regulator of invasion and metastasis [21]; the specific function of AKT2 in regulating the invasion process may be related to the specific phosphorylation targets of the kinase, such as mdm2, but may also be subject to cellular stresses such as oxidation which could uniquely activate AKT2 [22, 23].

Recently, we had previously shown that when AKT is activated in fibroblasts in organotypic rafts, there is a concomitant increase in KGF expression [5] implicating AKT signalling pathways in the regulation of KGF expression. Here we have shown that knockdown of all AKT isoforms in PTEN depleted fibroblasts was sufficient to reduce KGF expression and invasion. Interestingly, knockdown of individual AKT isoforms was not sufficient to disrupt the increase of KGF expression. This suggests there is intricate interplay between the different AKT isoforms and that loss of one isoform can be compensated for by the other family member, as has previously been observed in knockout animals [14] where individual knockout mice are viable; however, the AKT1 and AKT2 double knockout mice died *in utero* at day 18.5 and suffered from severe skin development [19]. While we have found that depletion of AKT2 was sufficient to abolish epithelial invasions mediated by PTEN depleted fibroblasts it did not alter KGF levels, suggesting that KGF-independent mechanisms are also able to regulate epithelial invasion.

This KGF-independent mechanism was shown to be due to a dramatic increase in IL1B expression, accompanied by enhanced secretion of IL1B. The enhanced secretion of IL1B was only observed in AKT2 depleted fibroblasts and was due to the increased expression of caspase 1 which is known to cleave IL1B resulting in secretion from cells. Whilst IL1B is known to drive KGF expression in fibroblasts [1] the effects of IL1B on the epithelium are not clearly defined. The IL1 isoforms, IL1A and IL1B, have previously been described as both anti-invasive and pro-invasive, suggesting no clear indication of their role in cancer progression [24-26]; however, our findings report a new protective function for IL1B in tumour invasion. We have demonstrated that treatment of AKT activated fibroblasts in organotypic cultures with recombinant IL1B inhibited epithelial cell invasion. This inhibitory role of IL1B was further confirmed by treatment of PTEN and AKT2 double knockdown fibroblast raft cultures with the naturally occurring negative regulator of IL1 signalling, IL1RA, which sequesters IL1B, resulting in restoration of epithelial invasion. Furthermore, examination of the oro-pharyngeal tumour samples at an mRNA level revealed that if IL1B is elevated in the stroma of the tumour compared to the stroma of matched non tumour tissue, patients have a more favourable outcome, than those where IL1B levels were reduced in the tumour samples. Previous datasets from microarray experiments support our findings as they reveal a favourable outcome for ovarian and breast cancer patients who express high levels of IL1B [27, 28]. The protective effect of IL1B in our model system, was due to the reduced expression of the KGF receptor, FGFR2b, by IL1B. Alterations of FGFR2B expression and single nucleotide polymorphisms have been implicated in a variety of cancers including breast, and gastric cancers [29-31] and here we show that the expression of FGFR2B correlates with the invasive behaviour of keratinocytes. This interplay between stromal fibroblasts and cancer cells has been shown in other cancers and in addition to regulating invasion, these interactions can change metabolism of tumour and stromal cells [32, 33].

In summary, our results show a crucial role for stromal AKT in regulating epithelial invasion, with AKT2 having an essential role in driving keratinocyte invasion from the stromal compartment. The adverse effects of AKT activation in the stroma can be counteracted by IL1B through down-regulation of the KGF receptor and so in cancer a balance between the pro-invasive AKT and KGF pathway and the anti-invasive IL1B levels within the stroma will drive the epithelium to either a more or less aggressive phenotype. The results suggest that AKT2 inhibitors may be effective in inhibiting invasion in oro-pharyngeal cancers

## MATERIALS AND METHODS

### Reagents

Total AKT (9272) and AKT p-473 (9271) antibodies were purchased from Cell Signaling Technology (Boston, MA, USA). FSP1 (sc-100784), FGFR2 (sc-122) antibodies and horseradish peroxidase conjugated secondary antibodies were purchased from Santa Cruz Biotechnology (Santa Cruz, CA, USA). pMDM2 (Ser 166) and beta-actin (AC74) antibodies were purchased from Sigma-Aldrich (St. Louis, MO, USA). The following sequences were used to produce pSUPER.puro (OligoEngine, Seattle, WA, USA) retroviral vectors for stable RNA interference: PTEN 5' TCC TGC AGA AAG ACT TGA AG 3'. shRNA targeting KGF was purchased from Origene (TR312988, puromycin selection, molecules 3 and 4)(Rockville, MD, USA). The following sequences were used for transient RNA interference: AKT1 5' GGA CGG GCA CAT TAA GAT CTT 3', AKT2 5' GAA GTG GCG GTC AGC AAGG 3', AKT3 5' GGC AAG AUG UAU AUG AUA A dT dT 3'. siRNA targeting total AKT (6211) was purchased from Cell Signalling Technology (Boston, MA, USA). Recombinant IL1B and IL1RA was purchased from Cell Signaling Technology (Boston, MA, USA). Akt1i and Akt2i were provided by Almac Discovery, Craigavon, Northern Ireland.

### Cell culture and stable/transient knockdown fibroblasts and immortalised keratinocytes

Primary Human Foreskin Fibroblasts, purchased from Cascade Biologics, and φnyx GP cells were maintained in Dulbecco's Modified Eagle Medium (DMEM) supplemented with 10% FBS, 1% Penicillin-Streptomycin solution and Amphotericin. Primary Human Foreskin Keratinocytes (HFK) were isolated as previously described [34] and maintained in Epilife media supplemented with Human Keratinocyte Growth Supplement (Invitrogen, Paisley, UK), 1% Penicillin-Streptomycin solution and Amphotericin. Organotypic raft cultures were generated as previously described [35] and cultured for 14 days, before being processed and Heamtoxylin and Eosin stained to standard protocols. Stable knockdown fibroblasts were generated using the φnyx system as described before [36]. Cells containing shRNA targeted towards PTEN were selected with 1.25 µg/ml puromycin. Cells containing shRNA targeted to KGF were selected using 3mg/ml Blasticidin. E6/7 keratinocytes were generated as previously described [37].

Transient knockdown was achieved by using 100 nM siRNA per 90mm dish of fibroblasts. siRNA was transfected using Polyethylenimine (PEI) at a ratio of 3µl to 100 nM siRNA. Fibroblasts were harvested 72 hours after transfection.

### Immunofluorescent staining and western blot analysis

Invasions were counted along the raft culture sections following H+E staining. Immunofluorescent staining of tumour samples was conducted as previously described [5], imaged using a DMI 6000B microscope and DFC-350 FX camera and visualised using LAS AF software (Leica Microsystems, Wetzlar, Germany). pAKT (Ser 473), AKT2, and FSP1 Antibodies were used at a concentration of 1:100, pMDM2 (Ser 166) antibody was used at concentration of 1:200 with an overnight incubation. For experiments shown in Figure 3D pMDM2 (Ser 166) antibody was conjugated using a fluorophore conjugation kit purchased from Biotium (Hayward, CA, USA).

Western blot analysis was performed as previously described [36]. Typically 50 µg of total protein was used to perform western blot analysis.

### Reverse-Transcription PCR analysis

RNA from cells and organotypic rafts was harvested using Trizol® (Life Technologies, Paisley, UK), while RNA from tissues was extracted using DNeasy Blood and Tissue kit (Qiagen, Crawley, UK) followed by Trizol extraction with linear acrylamide according to the manufacturer's instructions (Life Technologies, Paisley, UK). Reverse transcription was performed using M-MLV Reverse Transcriptase kit (Life Technologies, Paisley, UK). PCR analysis was performed using LightCycler® 480 SYBR Green I Master (Roche, Basel, Switzerland) according to manufacturer's guidelines. The following primer sets were used for analysis: *AKT1:* F: 5'-ATG ATG TGC GGT CGC CTG CC- 3'; R: 5'TGG GGC TTG AAG GGT GGG CT3', *AKT2:* F: 5'-AGT GGC GGT CAG CAA GGC AC-3'; R: 5'-TCA GCG CAG TGA GGA ACG GG-3', *AKT3:*F: 5'-TGG TGG GGC CTA GGG GTT GT-3'; R: 5'-TGG TCC TCC ACC AAG GCG TTT-3', *KGF:* F:5'-CAC AAA TGG ATA CTG ACA TGG-3';R: 5'-TCA CTC TTA TAT CCC CTC CTT C-3', *IL1A:* F: 5'-ATC CTT CTA TCA TGT AAG CTA TGG-3'; R: 5'-TCC TCT GAG TCA TTG GCG AT-3', *IL1B:* F: 5' TAC CTG TCC TGC GTG TTG AA 3'; R: 5' TCT TTG GGT AAT TTT TGG GAT CT 3', *FGFR2B:* F: 5' CCT GCC AAA ACA GCA AGC 3'; R: 5' AAG ACC CCT ATG CAG TAA ATG G 3'. The following controls were used *RPLPO:* F: 5'-ATC AAC GGG TAC AAA CGA GTC-3'; R: 5'-CAG ATG GAT CAG CCA AGA AGG-3'.

### ELISA analysis

IL1B ELISA was supplied by ebioscience (San Diego, CA, USA) and conducted according to the manufacturer's protocol. IL1B levels were quantified against a standard curve of recombinant human IL1B. Medium from organotypic raft cultures was concentrated 14 times before being applied to the ELISA.

## SUPPLEMENTARY METHODS


