# Supplementary material for: AKT in Stromal Fibroblasts Controls Invasion of Epithelial Cells
Source: Oncotarget. 2013 Jul 14;4(7):1103–16. doi: 10.18632/oncotarget.1078 (PMC3759669; doi:10.18632/oncotarget.1078)
Supplement: Supplementary file 1 [file oncotarget-04-1103-s001.pdf]

AKT in Stromal Fibroblasts Controls Invasion of Epithelial Cells - Cichon et al

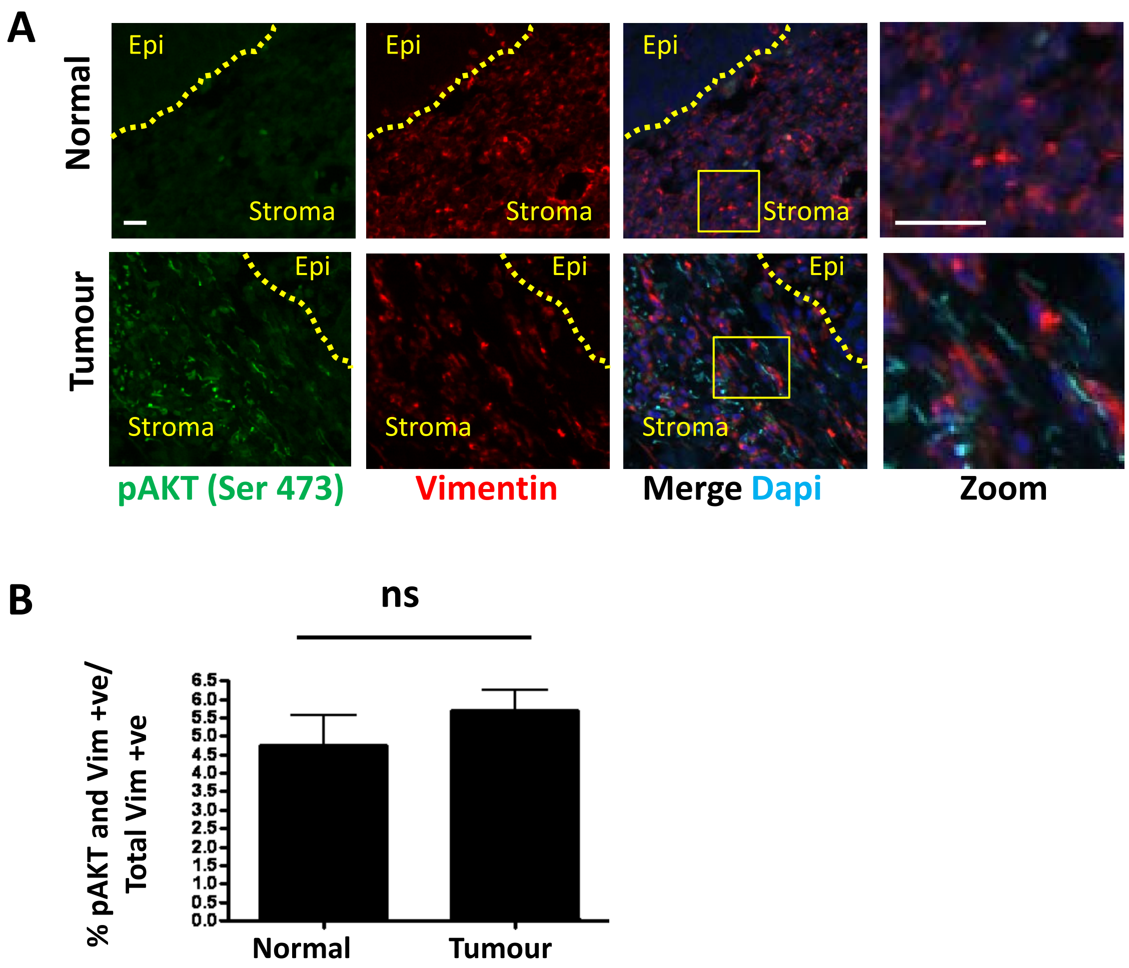

Supplemental Figure S1:

A) Oro-pharyngeal tumour samples were stained with anti-pAKT (Ser 473) and anti-vimentin. Scale bar = 25 µm. The boxed area is shown in the zoom panel. **B)** Quantification of staining, where cells were counted as follows: all vimentin positive cells were counted (to a total of 600 cells per normal and tumour section each per slide). Cells that dual stained for both pAKT (Ser 473) and vimentin were also counted. The histogram shows the percentage of cells that were pAKT(Ser 473) and vimentin positive out of all vimentin positive cells. All experiments were repeated on 3 occasions. ns=not significant.

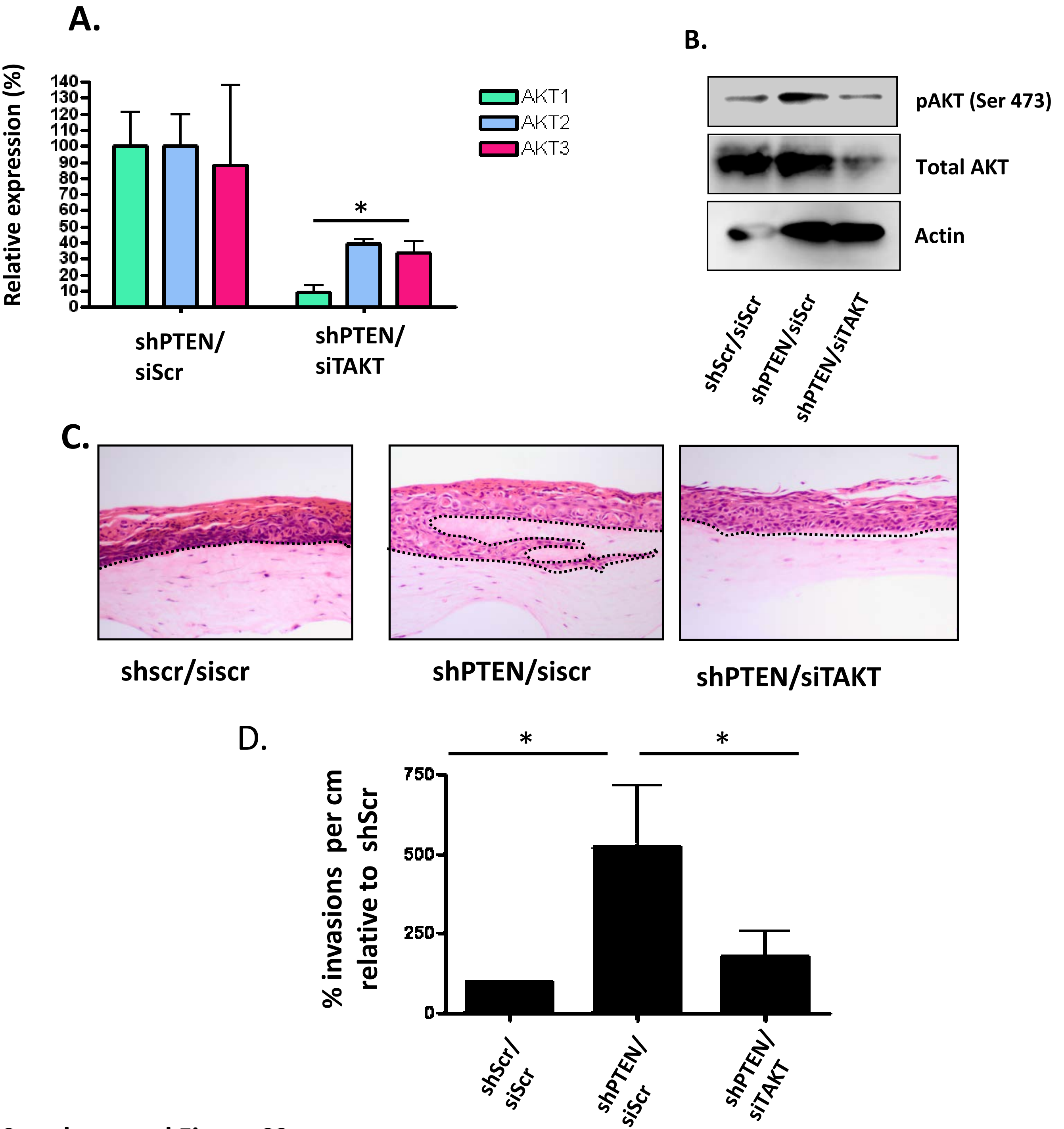

Supplemental Figure S2:

Confirmation of knockdown of all three AKT isoforms using a small interfering RNA targeting AKT1, 2 and 3 (siTAKT) by **A)** Real time PCR and **B)** Western blot. **C)** Organotypic raft cultures of scramble control (shScr/siScr), PTEN knockdown (shPTEN/siScr) and PTEN/TAKT (shPTEN/siTAKT) knockdown fibroblasts with E6/E7 expressing keratinocytes. Panels are representative of three separate experiments. **D)** Histogram shows quantification of invasions per cm of raft in relation to the scramble control raft culture shows an induction of invasions after stromal loss of PTEN and an inhibition of invasions upon combined PTEN and AKT knockdown. Average of three experiments.

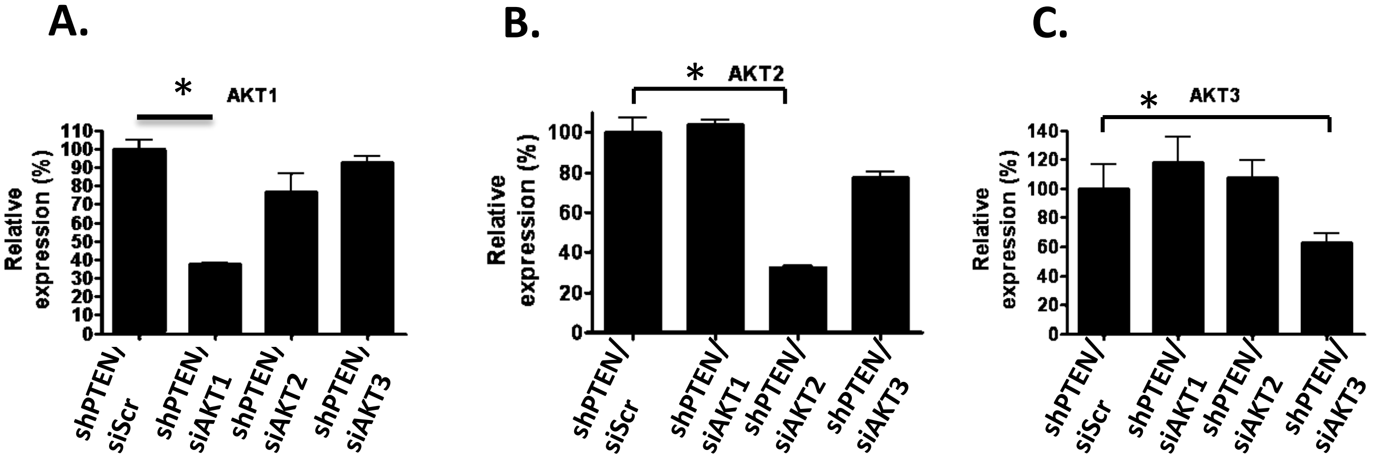

Supplemental Figure S3:

RNA was obtained from collagen plugs of PTEN and AKT depleted fibroblasts in organotypic raft cultures after 14 day culture. **A)** AKT1 **B)** AKT2 and **C)** AKT3 knockdown was determined by real-time PCR. P-values: * = $p<0.05$.

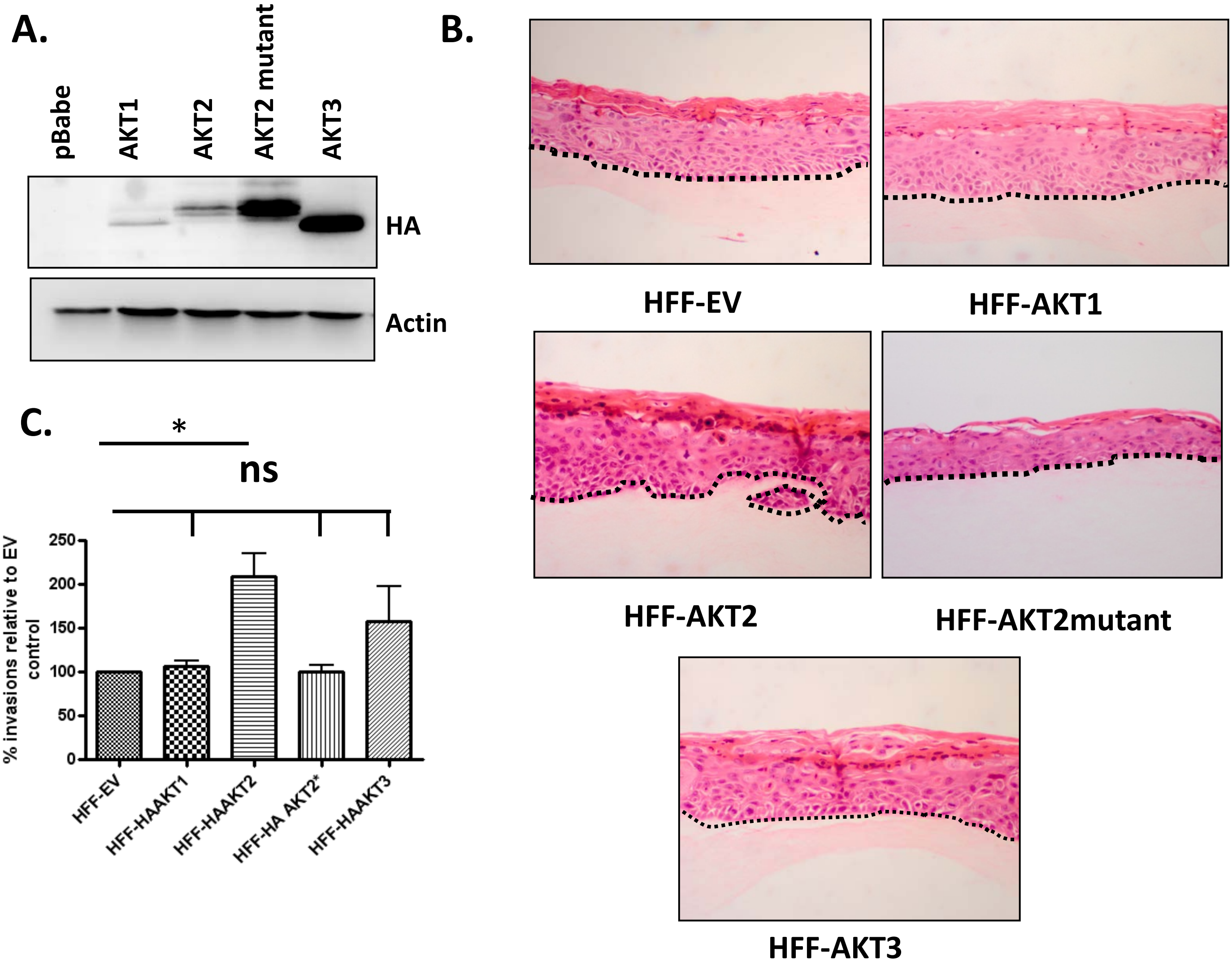

Supplemental Figure S4:

A) Western blot analysis of HA-tagged AKT isoform expression. Representative of three separate experiments. In all experiments the AKT2-mutant and AKT3 were expressed at a higher level than either the wild AKT1 or 2. **B)** Organotypic raft co-cultures using HA-tagged AKT isoforms or empty vector (EV) expressing HFFs and E6/E7 expressing keratinocytes. Representative of three separate experiments. **C)** Quantification of invasions per cm of raft culture relative to scramble control raft cultures. Quantification of three separate experiments. P-values: * = $p<0.05$. ns=not significant.

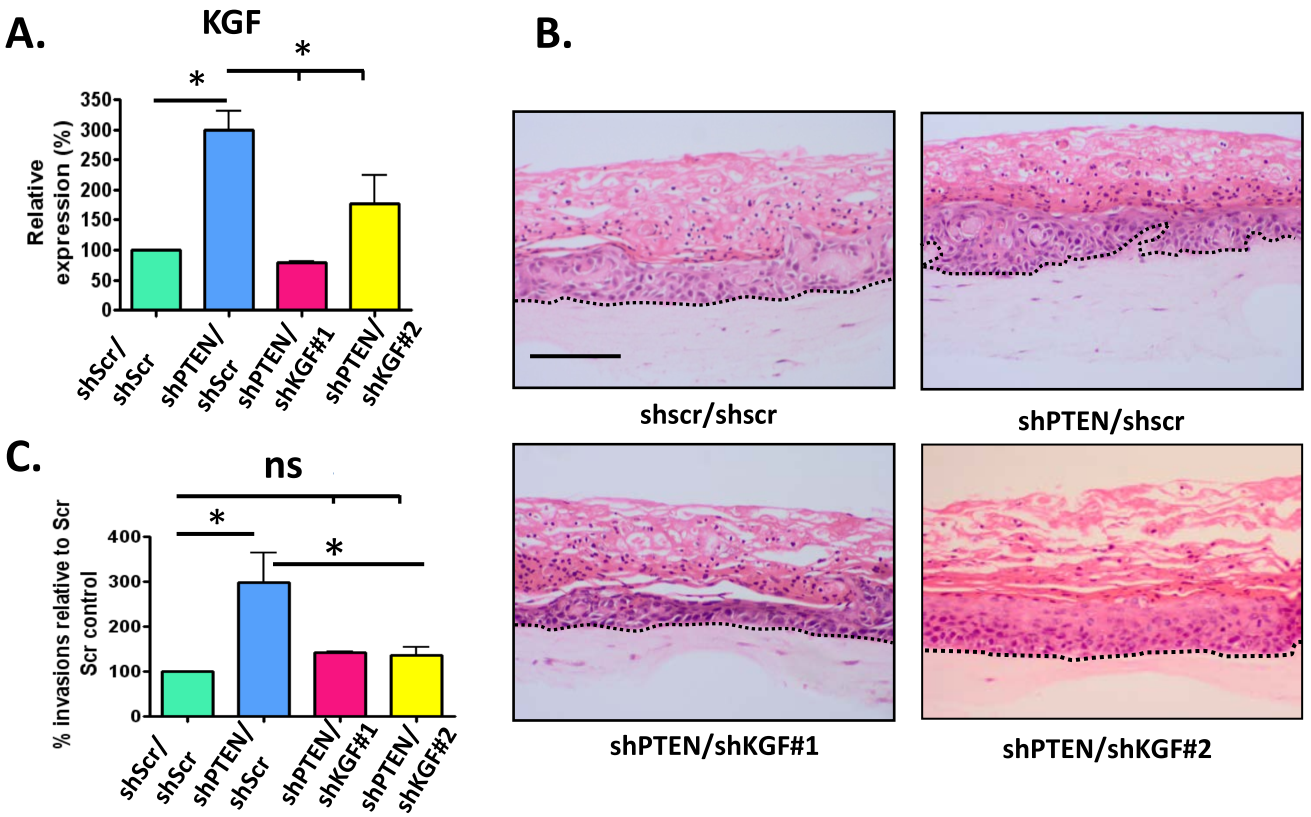

Supplemental Figure 5:

KGF mRNA levels in AKT depleted cells: **A)** Scramble control, PTEN and PTEN plus KGF knockdown fibroblasts were analyzed using real-time PCR analysis for KGF mRNA levels. **B)** Organotypic raft cultures of fibroblasts with scramble control (shScr), PTEN knockdown (shPTEN) and combined PTEN and KGF (shKGF＃1 and ＃2) knockdown cultured with E6/E7 expressing keratinocytes. Scale bar = 100μm. **C)** Quantification of invasions per cm relative to invasions occurring in scramble control raft cultures. P-values: *=p< 0.05. ns=not significant. All experiments were repeated 3 times. P values: *=p<0.05.

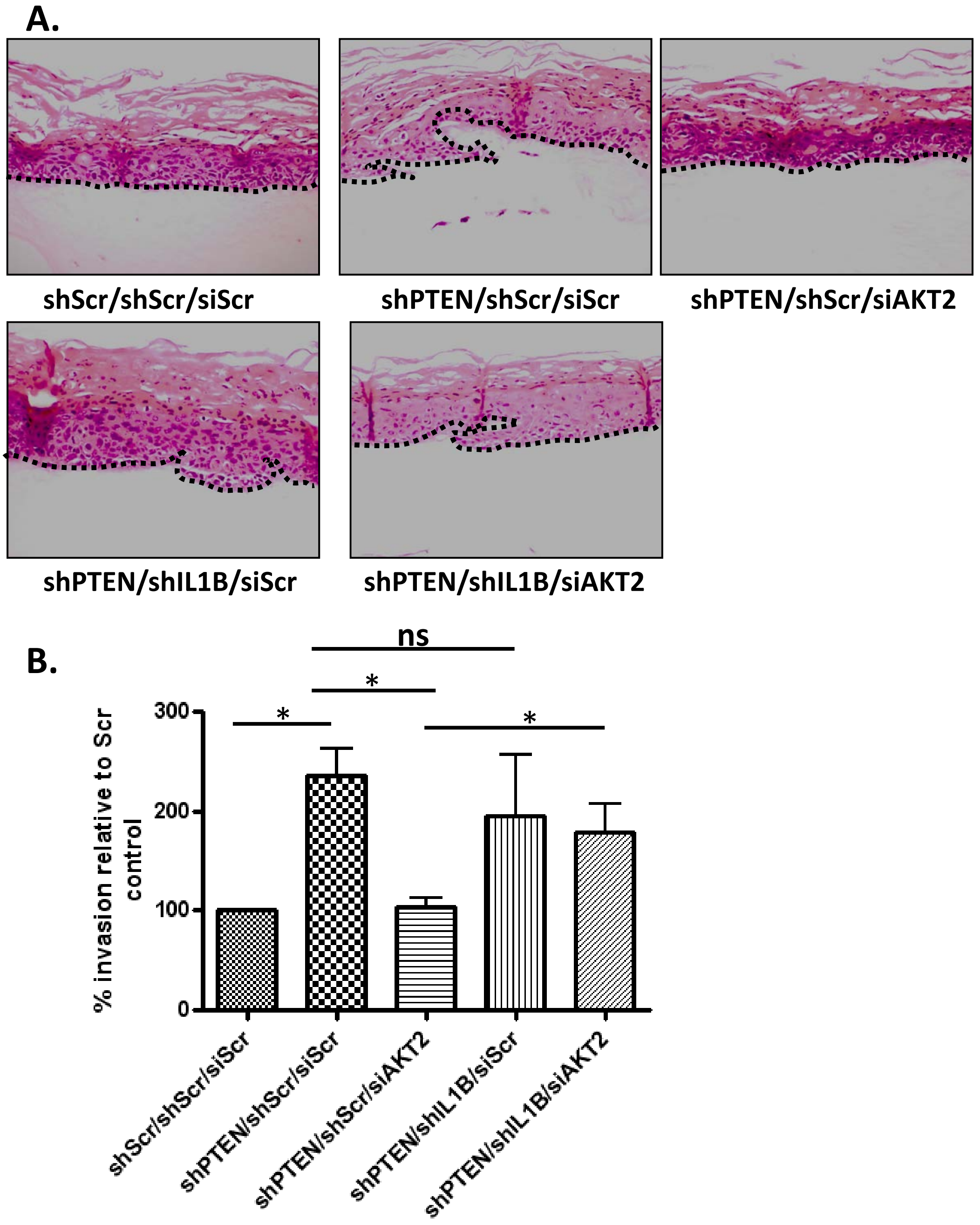

Supplemental Figure S6:

A) Primary human fibroblasts were depleted of PTEN, PTEN/AKT2, PTEN/IL1B and PTEN/AKT2/IL1B. Fibroblasts were used in organotypic raft co-cultures with E6/E7 expressing keratinocytes, representative of 3 experiments. B) Quantification of invasions into the collagen matrix per cm of raft culture relative to scramble control raft cultures from three independent experiments. P value: *=p<0.05. ns=not significant.
